# Efficient Middleware for the Portability of PaaS Services Consuming Applications among Heterogeneous Clouds

**DOI:** 10.3390/s22135013

**Published:** 2022-07-02

**Authors:** Salil Bharany, Kiranbir Kaur, Sumit Badotra, Shalli Rani, Kavita, Marcin Wozniak, Jana Shafi, Muhammad Fazal Ijaz

**Affiliations:** 1 Department of Computer Engineering and Technology, Guru Nanak Dev University, Amritsar 143005, Punjab, India; salil.bharany@gmail.com (S.B.); kiran.dcse@gndu.ac.in (K.K.); 2 Department of Computer Science and Engineering, Lovely Professional University, Jalandhar 144001, Punjab, India; sumit.26152@lpu.co.in; 3 Chitkara University Institute of Engineering and Technology, Chitkara University, Rajpura 140401, Punjab, India; shalli.rani@chitkara.edu.in; 4 Department of Computer Science, Engineering Chandigarh University, Mohali 140413, Punjab, India; kavita@ieee.org; 5 Faculty of Applied Mathematics, Silesian University of Technology, 44-100 Gliwice, Poland; 6 Department of Computer Science, College of Arts and Science, Prince Sattam bin Abdul Aziz University, Wadi Ad-Dawasir 11991, Saudi Arabia; j.jana@psau.edu.sa; 7 Department of Intelligent Mechatronics Engineering, Sejong University, Seoul 05006, Korea

**Keywords:** platform as a service, vendor lock-in, multi-clouds, middleware, platform services

## Abstract

Cloud providers create a vendor-locked-in environment by offering proprietary and non-standard APIs, resulting in a lack of interoperability and portability among clouds. To overcome this deterrent, solutions must be developed to exploit multiple clouds efficaciously. This paper proposes a middleware platform to mitigate the application portability issue among clouds. A literature review is also conducted to analyze the solutions for application portability. The middleware allows an application to be ported on various platform-as-a-service (PaaS) clouds and supports deploying different services of an application on disparate clouds. The efficiency of the abstraction layer is validated by experimentation on an application that uses the message queue, Binary Large Objects (BLOB), email, and short message service (SMS) services of various clouds via the proposed middleware against the same application using these services via their native code. The experimental results show that adding this middleware mildly affects the latency, but it dramatically reduces the developer’s overhead of implementing each service for different clouds to make it portable.

## 1. Introduction

The cloud computing paradigm has revolutionized the IT world with inherent advantages like scalability and cost savings. However, a few issues such as security and vendor lock-in, hinder its growth. The lack of interoperability and portability is the sole reason for vendor lock-in [1]. The platform-as-a-service (PaaS) layer constitutes the middle layer of the cloud computing stack, with the infrastructure-as-a-service (IaaS) layer being the bottom layer and the software-as-a-service (SaaS) layer the topmost layer. PaaS is the layer for software developers who need to use the services for their applications, and the provider is responsible for the infrastructure or the network for the operations. Application portability is the primary concern of the PaaS layer [1,2]. However, an application developed on one PaaS is not easily ported to another PaaS, leading to a situation known as vendor lock-in. The vendor lock-in problem is more prominent in the PaaS layer than in other layers. According to [3,4,5,6,7], the vendor lock-in situation, which is a consequence of the deficiency of interoperability among cloud vendors, refers to a “lack of ability to migrate application components and associated workloads from cloud provider A to cloud provider B.” Therefore, tackling vendor lock-in is very important; otherwise, using clouds can hinder the benefits and savings [1,2]. We propose a middleware class library developed in .NET Core to create an application that uses any of the supported services (message queue service, BLOB service, email service, and SMS) of any cloud (Google Cloud Platform, Amazon Web Services, and Microsoft Azure) and that can be easily ported into another supported cloud without any significant changes to the application source code [4]. A literature review was also conducted to gain insight into the approaches undertaken by researchers and the industry to deal with portability, especially application portability [5]. The solution approaches in the literature include standards, open libraries, common application programming interfaces (APIs), middleware, and model-based strategies. We chose the middleware approach as a solution for vendor lock-in as it is easy and efficient to integrate a middleware class library in new and existing applications.

### Application Portability among Interconnected Clouds

As there are numerous benefits of connecting clouds in various forms (federated clouds, multi-clouds, inter clouds, hybrid clouds) [2], applications developed targeting these collaborations also have inherent benefits:

(i) The ability to switch providers for better quality assurance if the provider cannot comply with the agreed service level agreement;

(ii) The ability to substitute another provider in case of an abrupt increase in the services’ prices or workloads;

(iii) Service providers may be selected in the heterogeneous geographical region due to legal constraints.

The authors of [2] categorized interconnected clouds into 25 categories. Interconnecting the clouds leads to the issue of interoperability and portability among heterogeneous clouds. Solving this issue of interoperability and portability breaks the vendor lock-in. There is some confusion about the interoperability and portability of clouds [1,3,4,5,6,7,8,9], and these terms are considered substitutes for each other, but [3,10] distinguished them. According to them, interoperability is defined as the capability of diverse systems to exchange information or work together seamlessly, whereas portability is the capability of moving a component from one provider to another without tampering with its usability. Portability is further divided into two types [11]:

(i) “Cloud data portability is the ability to easily transfer data from one cloud service to another cloud service, without being required to re-enter the data.”

(ii) “Application portability is the ability to easily transfer an application or application components from one cloud service to a comparable cloud service and run the application in the target cloud service [12,13,14,15,16,17,18,19].”

The focus of this paper is “application portability” and middleware to facilitate it. In addition, a survey of the different approaches to tackle this issue was conducted.

Defining “application portability” is essential before finding any solutions for it. Therefore, the literature was explored to retrieve the most appropriate definitions, which also revolve around PaaS portability [20,21,22,23,24,25,26,27,28] (although PaaS portability may involve much more than application portability). “Application portability” is the ease with which an application developed on one platform may be transferred and reused on another forum or different infrastructures within a cloud. In other words, a cloud-based application should be simply transferable and reused on another cloud system or infrastructure [29,30,31,32,33,34,35,36,37] (IaaS).”

## 2. Background and Related Work

The PaaS layer alleviates the developers’ burden of setting and maintaining the programming environment and the required infrastructure for the execution of an application [12]. However, there is considerable heterogeneity among disparate application platform offers, making it necessary to re-engineer an application before deploying it in a different environment [37,38,39,40,41,42,43]. A specific vendor provides proprietary services for developing applications, making them dependent upon the provider [13,14]. Moreover, one platform could supply some assistance that is not offered by another platform [10,43,44,45,46,47,48,49]. So, application portability among different clouds or from legacy enterprises to the cloud becomes essential to preclude users from vendor lock-in [50,51,52,53,54,55]. A vast amount of the literature on application portability solutions was reviewed, and a comparative analysis is presented in Table 1.

## 3. PaaS Cloud Application Portability Middleware

Although the portability of applications in the context of infrastructure-as-a-service (IaaS) is also possible with the help of virtual machines (VMs), mainly the portability of PaaS applications is covered in this article. Our objective here was to use the cloud-specific services so that the application remained portable without the intervention of the VM approach. We used .NET Core as our implementation platform, as it is open source and operating system-independent. Application portability does not only mean that the application is migrated or ported from one cloud to another cloud [21], although it is a possible case of application portability. It could involve the following scenarios, which our proposed solution is capable of handling:

(i) An application is migrated from Cloud A to Cloud B along with the application’s used PaaS services. Here, we assumed that Cloud B supported all the PaaS services used in Cloud A. This scenario can be classified as cloud-to-cloud portability.

(ii) The PaaS services used by the application are migrated from Cloud A to Cloud B, but the application remains on Cloud A. Either all PaaS services are ported to Cloud B, or some of the PaaS services are ported, depending on the PaaS services supported by Cloud B. In both cases, this migration falls under the classification of multi-cloud interoperability. 

(iii) The application is hosted on a private cloud (or another hosting service) but uses PaaS services from Cloud A and B. One PaaS service is finished from Cloud A, and the other PaaS service is destroyed from Cloud B. This scenario is an example of a hybrid cloud.

The authors of [12,32] identified possible incompatibility impediments when attempting the portability of a PaaS application from one cloud platform to another.

- Programming languages and frameworks: The programming languages supported by cloud platforms are limited. If an application developed in one platform using a specific language needs to be ported, that language should also be kept in the new cloud platform. The proposed middleware was created in the .NET Core framework for console and web applications. .NET Core is supported by most cloud platforms.
- Proprietary platform services: To facilitate developers in reducing the application’s development time, mainly the platform providers offer certain services via specific APIs. These services can be integrated into the applications by adding the cloud-specific software development kit (SDK) and implementing the platform-specific service by adding extra lines of code. Now there could be two possible scenarios:
  1. The specific service used by the application on one platform may not be available on the other.
  2. The new platform supports the specific service but offers a different interface.
    The proposed middleware mitigates these restrictions by allowing the user to keep using the service from the previous platform while the ported application is on the new platform.
- Data Storage: Database storage and file storage are required by most applications. There could be discrepancies due to different data storage types (e.g., SQL and NoSQL), additional data structures, and query languages. We covered data portability in another paper [41,42,43].
- Platform-specific configuration: Clouds use different variables to configure their platform services [44,45,46,47]. We defined the structure of our configuration file so that the middleware layer handles and implements the cloud services even with the different designs of the configuration variables. The configuration is not hardcoded in the middleware code [48,49,50,51]. Instead, it is provided in a JSON file that can be changed to alter the design even if the application runs. The middleware code does not need to be changed to facilitate the change in the configuration file. The modified configuration variables are loaded into the middleware directly via dependency injection [52,53,54].

### 3.1. Proposed Methodology Overview

This middleware is designed for .NET Core developers who want to develop a new application using PaaS services from the Microsoft Azure, Amazon Web Services, and Google Cloud platforms. It is also helpful for the developers who have already developed applications in .NET Core and want to integrate PaaS services into the existing application. Our library aims to provide application developers with an easy way to deploy their applications within different clouds without making any changes or making only minimal changes to the source code. Application migration becomes problematic when the developer uses any cloud-specific services, such as message queues, storage services, email services, etc., in their application. The difficulty arises when they need to migrate their application to another cloud. Now, either those services are not present in the cloud to which they are switching, or they have to make significant changes to their source code to accommodate the migration and functionality of the new cloud services.

**Figure 1:**
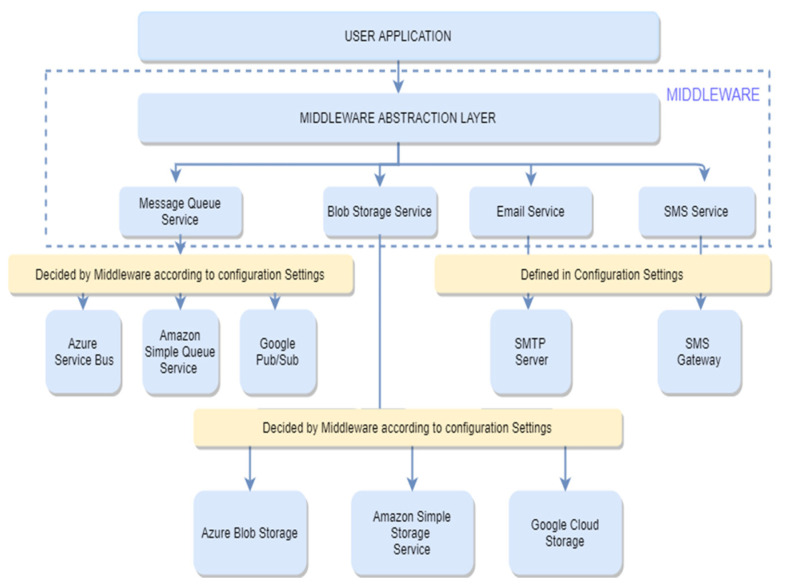
Architecture diagram of the proposed middleware.

We aimed to solve this problem by developing middleware (Figure 1) that supports various clouds and their services under one hood. The developer needs to specify the settings in the configuration file and is provided with an API to interact with our middleware abstraction layer to interact with the cloud services. In contrast, our middleware handles all the actual interactions with the cloud. The developer will interact with our middleware layer, and our middleware will further interact with the cloud service. This results in no changes to the source codes of developers’ applications, and they are free to move among the cloud platforms (Microsoft Azure, Amazon Web Services, and Google Cloud Platform). For every PaaS service of each cloud, the main issue for a common middleware library is that of creating a common configuration that can work for every cloud PaaS implementation without changing the source code in the middleware library as well as in the actual application in which the middleware library is going to be used. We developed the middleware and configuration file in such a way that developers do not need to change the source code for their applications in case they want to use another PaaS (supported by our middleware). They need to change the configuration file only and restart the application, and the application will adapt according to the new configuration file.

#### 3.1.1. Application Types Supported

Most legacy apps were developed using the monolithic approach, but nowadays, applications are produced using the microservices architecture. In a monolithic application, the complete functionality of a project exists in a single codebase. In contrast, microservices are an architectural pattern in which a single application is developed as a suite of small services. Each of these services runs in its process and communicates with a lightweight mechanism such as a HyperText Transfer Protocol (HTTP) resource API. Our middleware supports both the approaches, monolithic and microservices, and thus can be used for both kinds of applications. Although for the existing applications, some code changes are required to use our middleware, those are only one-time changes. After incorporating the changes, only minimal configuration changes are required if the developer wants to switch the data storage or other supported services.

#### 3.1.2. The Supported Services

The platform basic services provided by the PaaS platform providers and supported by our middleware are discussed in this section.

##### Email Service

Email service in our middleware is created using the classes named “MailMessage” and “SmtpClient” provided in the .NET Core framework. An email service made using these classes requires Simple Mail Transfer Protocol (SMTP) server settings in the application’s configuration file. The email service has a method named Send() that needs the object of EmailModel class defined in our middleware. The middleware handles the delivery of email using the SMTP settings provided in the application’s configuration file. Since email service uses SMTP client of .NET Core and not any cloud service, it becomes cloud-independent. Thus, it mitigates any special migration requirements for the cloud.

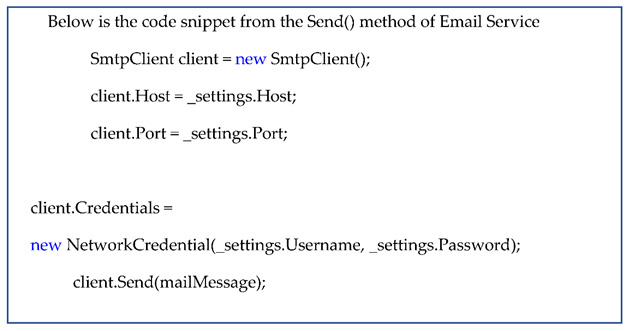



##### SMS Service

SMS service is configured to use a third-party SMS service known as Webaroo [1] (now known as GupShup). It provides a uniform resource locator (URL) that is hit to send an SMS. It requires a phone number, a message, and a GupShup account’s credentials. The developer is provided with a Send() method that requires a letter and a phone number to which the SMS will be sent. The credentials are provided in the configuration file. The service is bound to a single SMS service provider for now but can be enhanced to use with other SMS service providers. The following code snippet is an excerpt from the implementation of the SMS service:

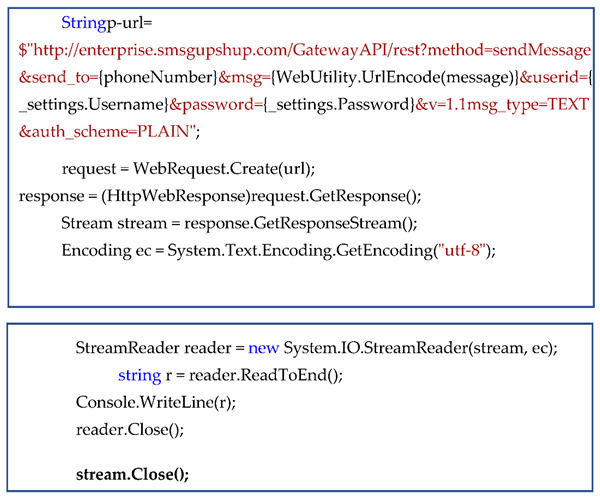



#### 3.1.3. Message Queue

A message queue is used to store messages used by applications to propagate messages between the presentation and business logic layers. It can be used for sending messages about tasks and orchestrating their execution. A message queue works in the first in, first out (FIFO) manner. The developer is provided with the middleware’s Send() and Receive() methods. The settings in the configuration file decide upon the platform’s message queue to be used: Azure Service Bus [1], Amazon Simple Queue Service (SQS) [2], or Google Pub/Sub4. The developer sets the settings in the application’s configuration file and interacts with the middleware using the abovementioned methods. Our middleware contains the implementation of the message queue services of all the three clouds and interacts with the relevant cloud according to the settings in the configuration file. If the developer changes the scenes from Azure to AWS, they do not have to change any code in their application. The middleware will detect the change in the configuration file and start using the message queue service of the new cloud. The developer will pass the message in the Send() method as shown below:

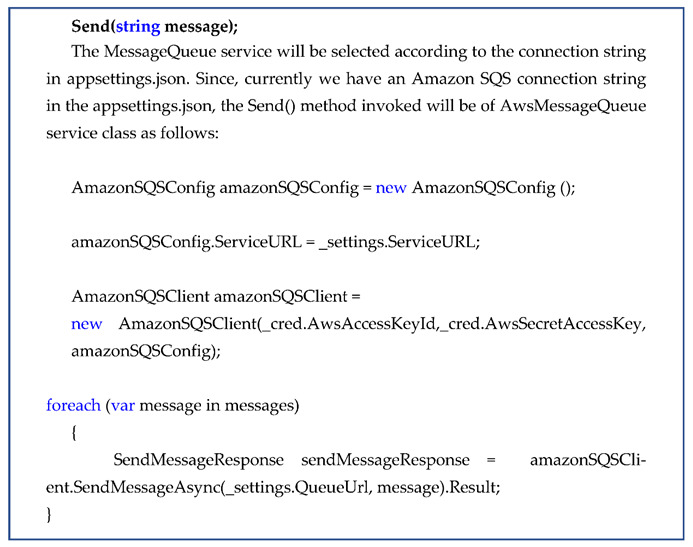



Similarly, we have also implemented Send () methods for Azure Service Bus and Google Pub/Sub.

#### 3.1.4. BLOB Storage

A BLOB storage system stores unstructured text and binary data as BLOBs that can be accessed by an HTTP(S) path. BLOB stands for Binary Large Object. It also provides security mechanisms to control access to data. Azure provides scalable, cost-effective, geo-redundant cloud storage, known as Azure Blob storage [1], for users’ unstructured data. Azure Blob storage handles trillions of stored objects for customers worldwide, with an average of millions of requests per second. Amazon Simple Storage Service [2] (Amazon S3) is an object storage service offering industry-leading scalability, data availability, security, geo-redundancy, and performance. This means that customers of all sizes and industries can use it to store and protect any amount of data for a range of use cases, such as websites, mobile applications, backup and restore, archive, enterprise applications, IoT devices, and big data analytics. Google Cloud Storage [3] is object storage for organizations that provide unlimited storage with no minimum object size, worldwide accessibility with low latency, and high durability. It also easily provides a transition to lower-cost tiers and storage classes for any workload. The developer is provided with the CreateFolder(), UploadFile(), ListFiles(), DownloadFile(), DeleteFile(), and DeleteFolder() methods of the middleware. These methods help them create/delete folders (Containers in Azure Blob Storage, Buckets in Amazon S3 and Google Cloud Storage) and upload/download/delete files (Blobs in Azure Blob Storage, Objects in Amazon S3 and Google Cloud Storage). For example, if the developer wants to upload a file, they need to call the following method:

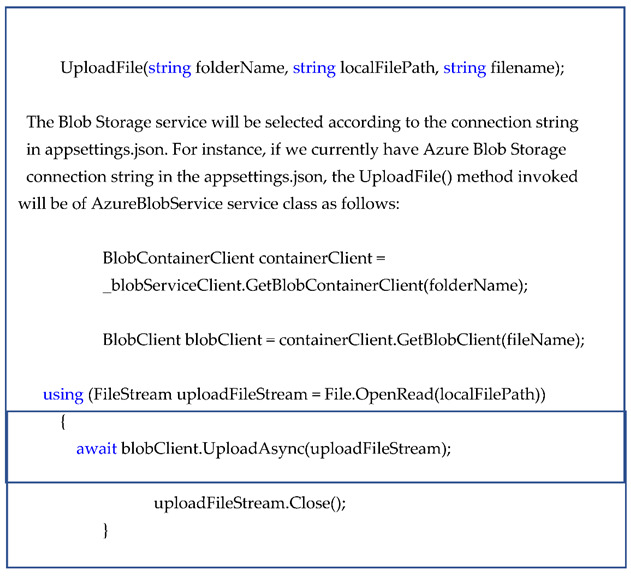



Similarly, we have implemented UploadFile() and other methods for Amazon S3 and Google Cloud Storage.

### 3.2. Implementation of Message Queue Service in Our Middleware

The developer needs to create an application using the .NET Core framework and add our middleware as a dependency package. Our middleware provides an interface named IMessageQueue that provides the developer with the Send() and Receive() methods. When the user passes a message to the Send() process, our middleware checks the cloud service used by reading the settings from the configuration file and loads the message queue service implementation of that specific cloud.

Algorithm 1 presents the pseudo-code for the implementation of the message queue service.

**Algorithm 1.** Pseudo-code for sending and receiving the messages from the message queue service.  //Method called by developer for sending messages
  MessageQueueService.Send(messages)
  
   //Implementation in Abstraction layer
  If cloud == ‘AWS’
settings = AwsSettings //From configuration file Create AwsMessageClient
  AwsMessageClient.Send(messages)
  
  If cloud == ‘Azure’
settings = AzureSettings //From configuration file Create AzureMessageClient
  AzureMessageClient.Send(messages)
  If cloud == ‘Google’
settings = GoogleSettings //From configuration file Create   GoogleMessageClient
  
  GoogleMessageClient.Send(messages)
  //Method called by developer for receiving messages
  messages = MessageQueueService.Receive(
  //Implementation in Abstraction layer
  If cloud == ‘AWS’
settings = AwsSettings //From configuration file Create AwsMessageClient
 return AwsMessageClient.Receive() //returns messages received from AWS cloud
  
If cloud == ‘Azure’
  settings = AzureSettings //From configuration file Create
  AzureMessageClient
      return AzureMessageClient.Receive() //returns 
  messages received from Azure cloud
  
      If cloud == ‘Google’
settings = GoogleSettings //From configuration file Create GoogleMessageClient
      return GoogleMessageClient.Receive() //returns 
  messages received from Google cloud

**Algorithm 2.** Pseudo-code for uploading and downloading a file to/from the BLOB storage service.  //Method called by developer for uploading file
 BlobService.UploadFile(fileName, folderName, localPath)
  //Implementation in Abstraction layer
   If cloud == ‘AWS’
settings = AwsSettings //From configuration file Create AwsBlobService
 AwsBlobService.UploadFile(fileName, folderName, localPath)
If cloud == ‘Azure’

settings = AzureSettings //From configuration file Create AzureBlobService
AzureBlobService.UploadFile(fileName, folderName, localPath)

If cloud == ‘Google’
settings = GoogleSettings //From configuration file Create GoogleBlobService
AwsBlobService.UploadFile(fileName, folderName, localPath)

//Method called by developer for downloading files
File = BlobService.DownloadFile(fileName, folderName, localPath)
//Implementation in Abstraction layer

If cloud == ‘AWS’
settings = AwsSettings //From configuration file Create AwsBlobService
return AwsBlobService.DownloadFile(fileName, folderName, localPath)
//returns messages received from AWS cloud
If cloud == ‘Azure’
settings = AzureSettings //From configuration file Create AzureBlobService
return AzureBlobService.DownloadFile(fileName, folderName, localPath) //returns messages received from Azure cloud

If cloud == ‘Google’
 settings = GoogleSettings //From configuration file Create GoogleBlobService
  return GoogleBlobService.DownloadFile(fileName, folderName, localPath) //returns messages received from Google cloud

Algorithm 2 presents the pseudo-code for the implementation of the BLOB storage service.

Then it sends a message to that specific cloud using the connection settings provided by the user in the configuration file. Similarly, when the Receive() method is called, the middleware checks the cloud service being used by the user’s application and loads the required message service [55,56]. Then it reads all the messages that are present in the cloud’s message queue and returns to the developer’s business logic layer, where they can perform their logic on the messages.

### 3.3. Implementation of Blob Storage Service in Our Middleware

**Figure 2:**
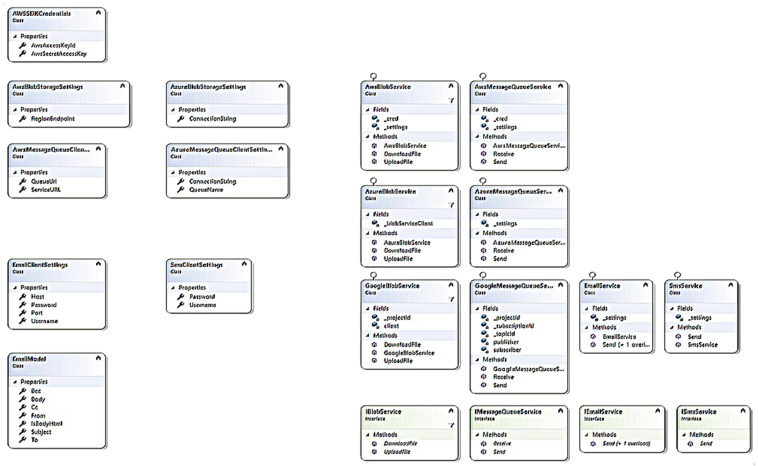
Class diagram for proposed methodology.

Our middleware provides an interface named IBlobStorage that provides the developer with UploadFile() and DownloadFile() methods. When the user passes a file to the UploadFile() method, our middleware checks the cloud service being used by reading the settings from the configuration file and loads the implementation of the BLOB storage service of that specific cloud. Then it uploads the file to that particular cloud using the connection setting provided by the user in the configuration file. Similarly, when the DownloadFile() method is called, the middleware checks the cloud service being used by the user’s application and loads the required BLOB storage service. Then it downloads the file . Class diagram of the proposed methodology can be seen in Figure 2.

## 4. Experimentation and Evaluation

We created two similar prototype applications in the .NET Core framework for the experimentation. We hosted the applications on Azure App Service. Azure App Service provides one free hosting with limited computing time. We used the free hosting slot. For implementing the platform services, one of the applications used the native code of the supported platform services, and the other application used our proposed middleware. The latency time was calculated as the time to perform the operations of send/receive messages by the message queue service, upload/download files in the BLOB storage service, send an email via the email service, and send an SMS via the SMS of the two applications. The overhead ∆ is defined as the ratio of the difference between the time taken to perform the various operations by the middleware and the native APIs and the time taken by the native APIs of the three clouds. To calculate the overhead percentage, we used the formula given in Equation (1).

(1) $$\Delta \;\%\;=\frac{\left(\mathrm{middleware}\;\mathrm{time}\;–\;\mathrm{native}\;\mathrm{time}\right)}{\mathrm{native}\;\mathrm{time}}\;\times \;100$$

**Figure 3:**
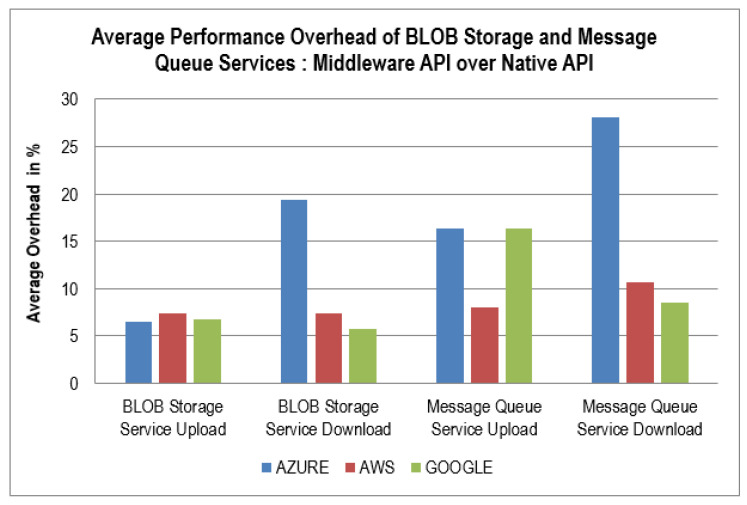
Performance overhead of BLOB storage service and message queue service.

**Figure 4:**
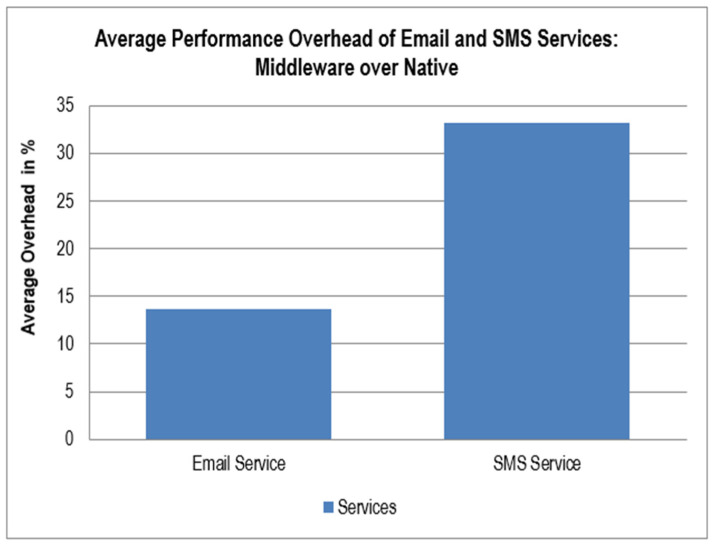
Performance overheads of email service and SMS service.

Table 2 and Table 3 shows the values of latency (in milliseconds) for the two applications using different services through the proposed middleware and the native code of various clouds. Figure 3 and Figure 4 report the graphs of the average performance overhead of these services. Our middleware performed extra operations compared to the native APIs of the PaaS services used, which led to a mild increase in the latency of the procedures performed. However, the overhead incurred by our middleware is justified due to the following reasons:

- Ease of use for the developer implementing the PaaS services in their application;
- Incorporating various PaaS services into a single middleware.

The middleware reads/writes the data in data stored in the native format and converts the fetched data into C# objects when brought into the code. This conversion of the data in C# objects leads to the ease of transformation of the data into various supported data storage formats.

The performance overhead graph (Figure 3) shows the average differences in the times taken in uploading/downloading 100, 500, and 1000 files (1MB each) to/from the BLOB storage and sending/reading 100, 500, and 1000 messages to/from the message queue on all the three clouds using native code versus using our proposed middleware. These average overhead percentages in both the implemented services are minimal except for the Azure cloud’s message queue “download” operation, thus proving that our middleware’s performance is optimal for real-world scenarios. If we consider the fact that the time taken to perform these operations is in milliseconds, then these times taken by the middleware and Azure cloud’s native API are extremely small (67, 43, 5, 9, and 7 milliseconds), and the differences among the values are also minimal. The performance of the middleware is quite close to that of the native API of the cloud. The efficiency of the BLOB storage service dramatically depends on the network stability of the user. The more stable the network is, the more efficient the BLOB storage service will be. For the BLOB storage service, the middleware’s implementation of the Azure cloud performed the best for the “file upload” operation. In contrast, Google Cloud performed with the most negligible overhead for the “file download” process.

**Figure 5:**
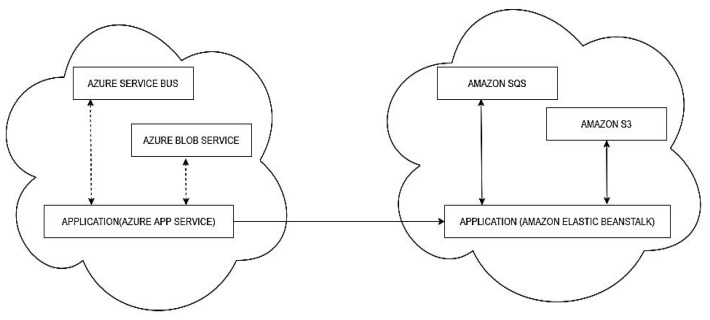
Cloud-to-cloud scenario.

A similar comparison is made in Figure 4 for email and SMS service in the sending of 10, 50, and 100 emails and 20, 100, and 200 SMSs using native code and our proposed middleware. The values of these overheads are also nominal and acceptable. Other portability scenarios were also successfully tested using the proposed middleware [43]. These scenarios were deployed with the following configurations (dashed lines show the previously supported clouds and services; solid lines show the new supported clouds and services):

Scenario (i): The application is hosted on Azure App Service and uses Azure Service Bus (message queue) and Azure Blob Storage. Then it is migrated to Amazon Elastic Beanstalk, and the PaaS services are migrated to Amazon Simple Queue Service (SQS) and Amazon Simple Storage Service (S3), as shown in Figure 5.

**Figure 6:**
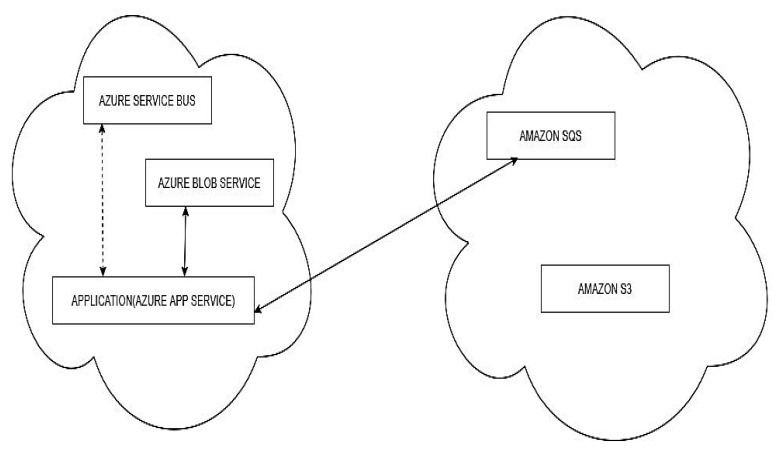
Multi-cloud scenario.

Scenario (ii): The application is hosted on Azure and remains there. The message queue service is migrated from Azure Service Bus to Amazon SQS. The BLOB storage service remains at Azure Blob Storage, as shown in Figure 6.

**Figure 7:**
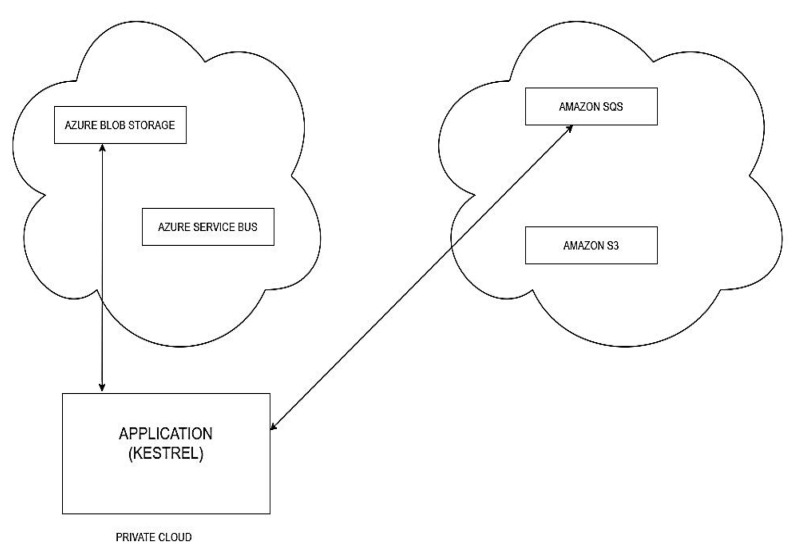
Hybrid cloud scenario.

Scenario (iii): The application is hosted on the local ASP.Net Core server (Kestrel). Amazon SQS is used for the message queue and Azure Blob Storage is used for the BLOB storage, as shown in Figure 7.

## 5. Conclusions and Future Work

The benefits of using clouds, such as cost savings, improved agility while managing the computing infrastructure, and the enhanced speed of system realization, lure consumers into migrating their services, data, and applications to a cloud. PaaS is the cloud computing paradigm layer where software developers primarily work on their applications as operations teams are responsible for infrastructure and hosting (IaaS layer). However, porting an application developed on one platform to another is not a trivial task. Middleware has been proposed in this paper to provide the developer with the facility to create a platform-independent cloud application. Microsoft Azure, Amazon Web Services, and Google Cloud Platform have currently supported cloud platforms. Developers can focus on their business logic rather than implementing the services for different clouds. Although the middleware does add mild latency to the services, it saves developers time by handling the implementations under the hood of abstraction of the services in the middleware. Our primary performance focus includes BLOB service and message queue service, which are commonly used cloud platform services. Email service can be implemented using the email functionality of the .NET Core framework. Every SMS provider has its own API, so creating a single generic solution is challenging. However, we implemented the SMS for one provider, i.e., Webaroo. Analysis of the significant work conducted on application portability was also performed in the study. We will be adding more cloud support (like Rackspace and IBM) and more PaaS services (such as Key Vault, Authentication, and Event Grid) so that developers will have more options to migrate. We will be optimizing the middleware so that its performance becomes as close as possible to that of the native code. We will also compare our approach with similar frameworks proposed in the literature.

## Figures and Tables

**Table 1:** Comparative analysis of solutions for application portability.

Reference	Approach	Focused	Technique Followed	Platforms Used	Tools Used for Implementation	Work Done
[15]	Cloud to cloud	A common set of PaaS providers’ capabilities	Categorization of PaaS portability problems	68 PaaS offerings	JSON	A standard architecture for heterogeneous platform-as-a-service platforms is proposed in this work to address the issue of application portability by identifying three layers: infrastructure, platform, and management.
[16]	Cloud to cloud	DevOps automation	Unified interface and adapters	Cloud Foundry, Heroku, CloudControl, OpenShift	RESTful API and a Ruby wrapper library	As a result of this article, the user may easily select the best cloud platform and manage and deploy cloud applications across several platforms.
[37]	Cloud to cloud	BLOB storage	Generic API and adapters	Microsoft Azure and Google App Engine	Jena API and SPARQL query language	An API that is semantically annotated for the automated construction of an adapter for a certain provider is proposed in this work.
[17]	Legacy to cloud	Cloud distribution of an application	Meta model	Sample application	JEE Café framework	In this article, a method for partitioning an app for cloud deployment (manually or with the help of optimization algorithms) is described.
[14]	Hybrid clouds	Flexibility of choosing a platform at deployment time rather than after deployment	Software architecture and security among applications’ different modules	-	-	Developing an application with certain components distributed on a cloud platform and others remaining on-premises raises several design difficulties, some of which are discussed in this article (on-premises).
[18]	Hybrid clouds	NoSQL storage, BLOB storage, and asynchronous task processing	Middleware (uniform API)	JBoss AS Cluster, GAE, RedHat, Openshift	JAVA APIs	Using middleware architecture, this study proposes a method for enabling hybrid cloud environments.
[19]	Cloud to cloud	Deployment, migration, and monitoring of applications	Abstraction layer	Cloud Bees, Cloud Foundry, Iron Foundry, Heroku	RESTful APIs	An abstraction of cloud providers’ differences in application deployment and lifecycle management is proposed in this study. An API was created by grouping together several core actions into a single set.
[35]	Cloud to cloud	Service-oriented architecture API	Standardized API	Private PaaS (PTIN Portugal Telecom Inovacao)	WSDL, SOAP/REST	Using industry-standard APIs, this paper outlines a distributed architecture for building and presenting services.
[20]	Cloud to cloud	Application and data portability	Semantic, model-driven, domain-specific language (DSL)	Android, Blackberry, Amazon EC2, GAE	Scalable Cloud Application Generator (SCALE), Modi Cloud	Semantics and domain-specific language (DSL) are leveraged for application portability in these studies from a user’s point of view.
[22]	Cloud to cloud	Integration of applications at PaaS level	Semantic and model-driven	-	Web Ontology Language (OWL) and DSL	The semantic technologies presented in this study serve as the foundation for a sophisticated application interoperability language.
[9]	Multi-cloud, legacy to cloud, cloud to cloud	Cloud-based application development	Common cloud API, semantic, and adapters	All major PaaS providers	RESTful implementation	To address the semantic interoperability challenges at the PaaS layer, this paper describes the Cloud4SOA project, which is built on a broker architecture.
[23]	Cloud to cloud, legacy to cloud	Application portability	Semantics and cloud patterns	Windows Azure	ODOL, OWL- S, SWRL	Cloud application portability is addressed in this study through the use of design principles and semantic technologies.
[24]	Cross-cloud (enterprise to cloud, enterprise to cloud to enterprise)	Storage, databases, and notification services	Common API and middleware	Google, Amazon, Azure, Rackspace	Java Persistence API (JPA), JAVA, XMPP, RESTful API	The service delivery cloud platform (SDCP) described in this article is a cloud middleware infrastructure that makes use of resources from a variety of different cloud service providers to deliver a wide range of services.
[40]	Cloud to cloud	Database	Containers	Amazon EC2 and Microsoft Azure	Runc Open Container, Flocker, Weave	The transfer of a Linux Container across a network is used for live application migration. Data and application states were tested across many cloud platforms in order to establish that they could be transferred across them.
[25]	Cross-cloud	Multi-cloud application deployment (virtual machines, network, storage)	Middleware software	Open Nebula, OpenStack clouds	Java, MySql, OVF format	Disparate cloud environments can be alleviated via the virtual execution platform (VEP) service, which this article describes. VEP automatically deploys the OVF packages on the IaaS clouds mentioned in the OVF files.
[26]	Cross-cloud, cloud to cloud	On-demand grouping of services of several clouds	Open source deployable cloudware (mOSAIC)	Most PaaS providers	Java, Python	European Union research project mOSAIC, a middleware framework for designing provider-agnostic, scalable cloud applications, is described in this article.
[33]	Legacy to cloud	Migration of different parts of an application	Cloud data patterns	Local company to cloud	-	This article discusses the process of moving on-premise software to the cloud, which necessitates various levels of re-engineering, depending on the kind of migration.
[39]	Cloud to cloud	Cloud-based application development	Software design patterns, API unification, adapters	Eucalyptus and OpenStack clouds	.NET, Java	This article discusses the process of moving on-premise software to the cloud, which necessitates various levels of re-engineering, depending on the kind of migration.
[34]	Cloud to cloud	NoSQL databases	Ontologies	Salesforce, Google App Engine (GAE), Microsoft Azure	Protégé, OWL, Resource Description Framework (RDF)	This research focuses on the semantic annotation of APIs and web services so that applications may be easily transferred across service providers. A variety of interoperability issues were discovered using a variety of ontologies and artificial intelligence (AI) planning.
[27]	Cloud to cloud	Customer resource management (CRM) software applications	MDE and DSL	-	AHEAD Composer, Eclipse framework	DSkyL, an Eclipse plugin that uses MDE for the building of customer relationship management (CRM) SaaS applications, is the subject of this publication.
[36]	Cloud to cloud	Monitoring cloud resources, storage accounts (BLOB, table, queue, etc.)	Model-driven engineering (MDE)	Microsoft Azure, GAE	-	In this research, a meta-model for cloud applications is provided that captures the essential elements of a cloud application.
[28]	Cross-cloud	Email, message queue, payment service	MDE (template-based approach) and code generation	Google, Amazon, Heroku	Eclipse framework, Xpand	An MDE-based approach is used in this study to make it possible to build cloud applications that can use services from several provider platforms at once.
[29]	Cloud to cloud	NoSQL	MDE and DSL	GAE and Microsoft Azure	Xtend, Xtext	Platform-independent DSLs are created in this study using MDE approaches. By utilizing this DSL, an application might be created that uses the specific cloud platform code.
[30]	Cloud to cloud	Discovery, transformation, and migration	MDE and DSL	IBM PaaS, GAE	MoDISCO, TXL	Using the model-driven architecture and refactoring technique, this article examines the high-level notion of an application’s migration between platform-as-a-service providers in three phases: discovery, transformation, and migration.
[31]	Cloud to cloud	SQL, BLOB, NoSQL, task queue, message queue, memcache, mailing	CPIM(a Java Library) and a common API	GAE and Microsoft Azure	Design Patterns (Abstract Factory Pattern)	PaaS-level services are encapsulated by a cloud provider independent model (CPIM) in this article to provide a mediation layer that hides the differences among multiple PaaS providers.
[13]	Hybrid and multi-cloud	BLOB storage service	MDE and adaptation	Microsoft Azure and Amazon S3 (Simple Storage Service)	Java, UML, XML, ATL, Maven	To generate platform-specific applications, the MULTICLAPP framework contains a transformation mechanism for mapping cloud artifacts to the target platforms.
[32]	Cloud to cloud, legacy to cloud	REST resources (message queues, object storage, etc.)	Abstraction and model-driven (DSL)	Microsoft Azure, Google, and AWS	Models, mapping, and generators	For a legacy or new application that uses REST APIs in the cloud, this article presents a hybrid strategy (abstraction and model-based) that would allow for the re-use of the same services on a different cloud.

**Table 2:** Values of latency times. Values of latency times for BLOB storage and message queue service.

Time Taken For		AZURE		Δ(%)	AWS		Δ(%)	GOOGLE		Δ(%)
		Middleware	Native		Middleware	Native		Middleware	Native	
BLOB Storage Service	100 files uploaded	251,206	235,784	6.54	134,892	125,673	7.34	412,879	386,791	6.74
	500 files uploaded	1,414,289	1,327,463	6.54	759,441	707,538	7.34	2,324,508	2,177,633	6.74
	1000 files uploaded	2,745,681	2,577,119	6.54	1,474,369	1,373,605	7.34	4,512,767	4,227,625	6.74
	Average Overhead			6.54	Average Overhead		7.34	Average Overhead		6.74
	100 files downloaded	60,879	53547	13.69	61,505	57,296	7.35	200,543	189,562	5.79
	500 files downloaded	342,748	301,469	13.69	346,273	322,576	7.35	1,129,057	1,067,234	5.79
	1000 files downloaded	765,407	585,268	30.78	672,249	626,245	7.35	2,191,934	2,071,912	5.79
	Average Overhead			19.38	Average Overhead		7.35	Average Overhead		5.79
Message Queue Service	100 messages uploaded	13,967	12,846	8.73	10,484	9627	8.90	6135	5683	7.95
	500 messages uploaded	62,412	60569	3.04	48,783	44,200	10.37	2500	1995	12.71
	1000 messages uploaded	116,025	84406	37.46	110,353	105,223	4.88	3999	3115	28.38
	Average Overhead			16.41	Average Overhead		8.05	Average Overhead		16.34
	100 messages downloaded	67	43	55.81	1574	1457	8.03	7575	6721	12.71
	500 messages downloaded	5	5	0.00	6659	6202	7.37	6721	6439	5.86
	1000 messages downloaded	9	7	28.57	18,594	15,925	16.76	6393	5980	6.90
	Average Overhead			28.12	Average Overhead		10.72	Average Overhead		8.49

**Table 3:** Values of the Latency Times for Email and SMS service.

Time Taken For		Middleware	Native	Δ(%)
Email Service	10 emails sent	155,367	136,127	14.13
	50 emails sent	808,640	693,836	16.55
	100 emails sent	1,560,560	1,415,639	10.24
		Average Overhead		13.64
SMS Service	20 SMS sent	6512	5286	23.19
	100 SMS sent	37,430	25,540	46.55
	200 SMS sent	69,165	53,190	30.03
		Average Overhead		33.25

The values of the latency times mainly depended on the user’s network speed, location of the cloud data center, and configuration of the server where the application was hosted.

## Data Availability

Not applicable.
